# Clinical advances and multifactorial pathophysiological mechanisms of neuropsychiatric comorbidity in systemic sclerosis

**DOI:** 10.3389/fimmu.2026.1838407

**Published:** 2026-07-10

**Authors:** Jiaxin Chen, Ronghong Guo, Xianzhe Zhou, Jinfang Gao, Ke Xu

**Affiliations:** Third Hospital of Shanxi Medical University, Shanxi Bethune Hospital, Shanxi Academy of Medical Sciences, Tongji Shanxi Hospital, Taiyuan, China

**Keywords:** immune, neuropsychiatric comorbidity, pathophysiological mechanisms, systemic sclerosis, therapeutic implications

## Abstract

This review addresses the high prevalence of depression and anxiety in systemic sclerosis (SSc), as well as the core biological, psychological and social mechanisms underlying such psychiatric comorbidities, The prevalence of clinically significant depression or anxiety disorders reaches 30%–50% among patients with SSc who satisfy corresponding diagnostic criteria. Established risk factors encompass multi-organ involvement (e.g., lung fibrosis, digital ulcers), chronic pain, physical disability and disease-related stigma. Three key pathophysiological mechanisms drive this comorbidity: immune dysregulation induces neuroinflammation via pro-inflammatory cytokines (IL-6, TNF-α), pathogenic autoantibodies and gut-brain axis disruption; cerebral microvascular damage and chronic hypoxia impair emotion-regulating neural circuits; tissue fibrosis and persistent stress over activate the HPA axis, forming a pathogenic cycle connecting systemic inflammation, glucocorticoid resistance and negative mood. Clinical management of SSc should extend beyond conventional antidepressants and cognitive behavioral therapy to include integrated rheumatology-psychiatry care, IL-6/JAK-targeted biologics and transcutaneous vagus nerve stimulation (tVNS), all designed to ameliorate patients’ physical and psychiatric symptoms simultaneously. In conclusion, psychiatric comorbidity in SSc stems from the intricate interplay of biological, psychological and social factors. Elucidating these mechanisms facilitates the development of targeted therapeutic strategies that address both systemic SSc manifestations and associated mental health conditions, thereby enhancing patients’ health-related quality of life and long-term psychiatric outcomes.

## 1 Introduction

SSc is a chronic systemic autoimmune rheumatic disease characterized by skin and organ fibrosis, microvascular damage, and immune dysregulation (1). Skin thickening, the earliest and most recognizable clinical feature of systemic sclerosis, defines two distinct cutaneous subtypes: limited cutaneous SSc (lcSSc) and diffuse cutaneous SSc (dcSSc). These subtypes differ in the extent of skin involvement, risk of visceral organ complications, and overall prognosis (2). SSc also commonly affects the lungs, heart, kidneys, and gastrointestinal tract (3), leading to a high comorbidity burden and premature mortality. Depression symptoms, anxiety symptoms and somatization is higher in patients with SSc than in healthy individuals (4, 5). These conditions worsen disease burden, reduce treatment response and quality of life, and increase disability and mortality risk (6). SSc and mental disorders share overlapping pathogenic mechanisms. Key pro-inflammatory cytokines (IL-1β, IL-6, and TNF)drive immune dysregulation in both conditions (7, 8). Critically, SSc-specific vascular pathology and progressive fibrosis of skin and internal organs are not merely parallel features: robust clinical evidence links them directly to elevated incidence and worsening course of depression and anxiety disorders (9). Furthermore, bidirectional interactions exist: the chronic pain caused by Raynaud phenomenon and digital ulcers in SSc patients can induce or exacerbate sustained activation of the stress response and subsequent release of pro-inflammatory cytokines; at the same time, SSc patients may develop mental disorders due to gastrointestinal involvement, fatigue, appearance-related distress, immune abnormalities, and corticosteroid therapy (10). Conversely, mental disorders impair immune regulation and reduce treatment adherence, thereby accelerating SSc progression and diminishing therapeutic response.

SSc has a complex, multifactorial etiology and highly heterogeneous clinical presentation. Key symptoms (including fatigue, cognitive dysfunction, and autonomic or central nervous system involvement)frequently overlap with manifestations of depression, anxiety, and other psychiatric disorders. This symptomatic convergence impedes timely recognition by both patients and clinicians, contributing to underdiagnosis and delayed psychiatric intervention. Additionally, the use of corticosteroids for the treatment of systemic sclerosis may induce a variety of psychiatric symptoms including anxiety, depression, psychosomatic disorders, cognitive impairment and disorientation (11). Therefore, systematic screening, prompt diagnosis, and early intervention for mental disorders in SSc patients are clinically essential—not only to mitigate psychiatric morbidity but also to improve disease control, treatment adherence, functional status, and overall quality of life. This article focuses on how SSc-associated fibrosis, microvascular damage, and neuro-immune crosstalk collectively impair mental health. It further highlights translational implications and offers novel mechanistic insights that inform strategies to improve quality of life and advance targeted, integrative therapeutic approaches for SSc.

## 2 Epidemiology and clinical manifestations of SSc psychiatric disorders

Approximately 50% of SSc patients experience psychiatric symptoms ranging from mild to severe (12). Depression prevalence is markedly elevated in SSc: a large-scale epidemiological study by Bragazzi et al. reported significantly higher rates compared with healthy controls (13), and this finding was replicated in a longitudinal cohort of 3,270 SSc patients (6). Using the Hospital Anxiety and Depression Scale (HADS), Garaiman et al. found high symptom burdens in 316 SSc patients: 32.2% met criteria for anxiety, 25.9% for depression, 18.5% for mixed anxiety–depression disorder (MADD), and 49.5% for psychological distress (14). All exceeded population norms (15). Furthermore, sleep disturbances affect over 70% of patients, and approximately 61% exhibit mild cognitive impairment (16, 17). Obsessive–compulsive disorder affects approximately 15% of individuals with SSc (18). The researchers adopted the Mini International Neuropsychiatric Interview (MINI) to assess mental disorders among patients with SSc. The prevalence of panic disorder characterized by recurrent, unexpected panic attacks featuring palpitations, sweating, dyspnea, derealization or depersonalization, nausea, abdominal discomfort, or a sense of impending death (19),was 11.5% in the SSc group, versus 4% in control subjects (3).

Psychiatric manifestations in SSc are highly heterogeneous, encompassing affective, behavioral, and autonomic symptoms. Core depressive features, including anhedonia, excessive self-blame, and guilt, are relatively specific to SSc-related mood disturbance, whereas fatigue, insomnia, and unintentional weight loss are highly prevalent nonspecific symptoms (20). These manifestations arise from multiple interacting factors: the diagnostic and prognostic uncertainty of SSc, progressive organ involvement, socioeconomic strain, and functional impairment. Anxiety disorders, which are understudied yet clinically significant in SSc, commonly present with persistent worry, restlessness, sleep disruption, fatigue, irritability, diaphoresis, and tremor (21). Potential contributors include visible disfigurement due to skin sclerosis and disease unpredictability (10). Obsessive–compulsive disorder (OCD) is defined by intrusive, distressing thoughts and compulsive rituals (22).

In clinical practice, mental health assessment in SSc patients routinely begins with validated self-report scales (**Table 1**), which provide an initial, symptom-based screening of psychological status. For definitive diagnosis, these scales are integrated with the MINI, and diagnoses are established according to ICD-11 or DSM-5 criteria. They support reliable identification of depressive disorder, anxiety disorders, and other common conditions. In contrast, disorders such as OCD rely primarily on structured clinical interviews and comprehensive longitudinal history, given their lower sensitivity to brief screening instruments and greater dependence on phenomenological detail and functional impact.

**Table 1:** Standardized assessment scales for common mental disorders.

Questionnaire	Content	Reference
PHQ-9	It assesses both emotional and somatic symptoms associated with depression, such as feelings of hopelessness, diminished interest, and loss of appetite.	(23)
CES-D	It is designed to assess depressive affective symptoms experienced during the previous week.	(24)
BDI	It comprises items related to depressive symptoms, encompassing hopelessness, irritability, and guilt, as well as somatic symptoms such as fatigue, weight loss, and diminished interest in sex.	(25)
GAD-7	The scale contains questions focusing on the frequency of anxiety symptoms over the past two weeks, with additional utility in assessing panic disorder, social anxiety disorder, and post-traumatic stress disorder (PTSD).	(19)
PSQI	It provides a multidimensional assessment of sleep quality through 24 items, It is the most widely utilized instrument for subjective self-reported sleep assessment.	(26)
SCL-90-R	This instrument is designed to concurrently assess nine different symptom domains, encompassing somatization, obsessive-compulsive tendencies, interpersonal sensitivity, depression, anxiety, phobic anxiety, hostility, paranoid ideation, and psychoticism.	(27)

PHQ-9, the Patient Health Questionnaire; CES-D, the Centre for Epidemiologic Studies Depression Scale; BDI, Beck Depression Inventory; GAD-7, Generalizede Anxity Disorder-7; PSQI, Pittsburgh Sleep quality index; SCL-90-R, Symptom Checklist 90-R.

## 3 Bidirectional interactions between somatic manifestations and psychiatric disorders in SSc

Pain, sleep disturbances, fatigue, pulmonary involvement and skin fibrosis are common complications of systemic sclerosis.

### 3.1 Complications of pain and sleep disturbances

Pain is among the most prevalent and disabling symptoms in SSc. Epidemiological data indicate that over 87% of patients experience at least mild pain, 38% report moderate-to-severe pain, and approximately 35% require targeted pain interventions (28, 29). Joint pain is the most common pain phenotype in SSc, alongside manifestations such as Raynaud’s phenomenon, digital ulcers, digestive tract pain, and polyneuropathy (30). Pain exhibits robust bidirectional associations with depression, anxiety, and sleep disturbances. Relative to the general population, individuals with chronic pain face a heightened risk of emotional distress and anxiety symptoms (31). Racine M et al. investigated the impact of pain on functional status and health-related quality of life (HRQL) in SSc patients, demonstrating that pain exerts a significant negative influence on multiple functional domains, including depressive symptom severity, fatigue burden, and global disability scores. Further subgroup analyses revealed a dose-dependent relationship: SSc patients with higher self-reported pain intensity exhibited more severe depressive symptoms (32). Pain is also a core precipitant of sleep disorders, which affect 63–94% of SSc patients, with rates substantially higher than in healthy controls. Objective sleep abnormalities include reduced sleep efficiency, diminished rapid eye movement (REM) sleep duration, elevated arousal indices, and amplified slow-wave sleep activity (16, 33). A separate longitudinal study confirmed a bidirectional link between sleep disturbances and anxiety: individuals with sleep disorders had a 1.89-fold increased risk of developing anxiety symptoms, while pre-existing anxiety augmented the risk of sleep disorders by 1.2-fold (34).

Collectively, these findings support the existence of a complex, mutually reinforcing symptom network in SSc, where psychiatric disorders, sleep disturbances, and pain perpetuate each other’s severity.

### 3.2 Complications related to fatigue

Fatigue affects approximately 75% of patients with SSc, a prevalence that markedly exceeds that of healthy controls (35). When persistent and meeting the criteria for chronic fatigue, it serves not only as a core disease-related symptom but also as a significant risk factor and comorbid driver of psychiatric morbidity. In adult SSc cohorts, up to 72% of individuals who report clinically significant fatigue concurrently meet standardized diagnostic criteria for at least one psychiatric disorder, including depressive disorder (40–47%), anxiety disorders (32%), and somatic symptom disorder (15%). Critically, evidence supports a bidirectional pathophysiological relationship between fatigue and psychiatric disorders. Persistent fatigue may promote psychiatric vulnerability through behavioral mechanisms, such as reduced engagement in rewarding or physically activating activities, impaired social role functioning, and diminished HRQL. Conversely, psychiatric disorders can intensify fatigue via neurobiological pathways, sleep architecture disruption, and altered frontostriatal motivational circuitry (36).

### 3.3 Complications of pulmonary involvement

Respiratory complications are the leading cause of mortality in SSc (37). Interstitial lung disease (ILD) is the most prevalent pulmonary manifestation, affecting approximately 30% -50% of SSc patients (38). Its core clinical features include dyspnea, chronic cough, and nonspecific interstitial pneumonia (39). Pulmonary arterial hypertension (PAH), a distinct yet equally life-threatening complication, occurs in 10% of SSc patients (40). It is characterized by exertional dyspnea, syncope, and hemoptysis. Robust epidemiological and longitudinal evidence consistently links respiratory involvement to elevated risks of psychiatric morbidity (41). A multivariate analysis of depression predictors in SSc identified chronic hypoxemia as an independent correlate of depressive symptom severity (42). In ILD cohorts, 31% meet DSM-5 criteria for anxiety disorders and 23% meet criteria for MDD. Among SSc-PAH patients, psychiatric comorbidity affects >35%, with MDD as the most frequent diagnosis. Notably, the prevalence of MDD in this subgroup is threefold higher than in the general population (43).

### 3.4 Complications of skin fibrosis

Cutaneous fibrosis in SSc, particularly facial skin tightening and hand flexion contractures, induces clinically meaningful body image disturbance. This disturbance is associated with impaired social functioning, behavioral avoidance, and an elevated risk of social anxiety disorder (44). It correlates strongly with hopelessness, a core cognitive feature of major depression, and may therefore serve as a proximal psychological mediator in the pathogenesis of depressive disorders in SSc (45). Furthermore, dissatisfaction with facial appearance and greater severity of esophageal and gastrointestinal involvement are independently associated with heightened state and trait anxiety symptoms (9). Psychosocial stressors, including household income reduction, erosion of perceived social support, and job loss, are robustly linked to psychiatric comorbidity in SSc (46). These stressors frequently arise secondary to disease-related functional limitations: restricted mobility, chronic fatigue, persistent pain, and hand dysfunction impair occupational performance and social participation, thereby amplifying vulnerability to mood and anxiety disorders.

## 4 Biological mechanisms underlying comorbid mental disorders in SSc

### 4.1 Immune dysregulation

Immune dysregulation is a hallmark pathological feature of SSc, and immune abnormalities are also frequently observed in patients with psychiatric disorders (47). Psychiatric disorders are closely linked to the morbidity and severity of autoimmune diseases including systemic lupus erythematosus(SLE) (48), multiple sclerosis (MS) (49), and rheumatoid arthritis (RA) (50). Studies on disease activity assessment in patients with spondyloarthritis and Sjögren’s syndrome also demonstrate that psychiatric disorders can raise the risk of comorbidities and exacerbate disease activity (51, 52).

#### 4.1.1 Adaptive immunity

Based on functional specialization, T cells are categorized into helper T lymphocytes (Th), cytotoxic T lymphocytes (CTL), and regulatory T cells (Treg).

In SSc, the Th1/Th2 immune balance is skewed toward Th2 polarization, driving excessive secretion of cytokines including IL-4 and IL-13. Notably, IL-4 directly induces collagen synthesis in fibroblasts, while IL-13 acts as a potent profibrotic mediator to accelerate fibrotic progression (53). Concurrently, the frequency of Th17 cells is elevated in both the peripheral blood and lesional tissues of SSc patients; their secreted IL-17 not only triggers autoimmunity and amplifies local inflammation but also directly promotes fibrotic remodeling. Furthermore, SSc patients exhibit reduced Treg cell proportions and functional impairment, which leads to diminished production of the anti-inflammatory and antifibrotic cytokine IL-10, alongside aberrant secretion of Th2-type cytokines (IL-4 and IL-13)—a cascade that further exacerbates tissue fibrosis (54).

Immune dysregulation is a well-established pathogenic driver across multiple neuropsychiatric disorders. In MDD, Th1 cell expansion drives elevated secretion of TNF-α, IFN-γ, and IL-2, which collectively activate microglial indoleamine 2,3-dioxygenase (IDO) and kynurenine 3-monooxygenase (KMO). This enzymatic activation shifts the kynurenine pathway toward quinolinic acid accumulation, which suppresses brain-derived neurotrophic factor (BDNF) expression and exacerbates neuroexcitotoxicity, thereby contributing to core depressive phenotypes including anhedonia, psychomotor retardation, and executive dysfunction (55). In schizophrenia, diminished Treg frequency is robustly associated with negative symptom burden and deficits in working memory and attentional control (56). Conversely, Th17 cell expansion promotes sustained neuroinflammation via IL-17A and IL-6, inducing neuronal hyperexcitability, impairing long-term potentiation, and promoting apoptosis—processes mechanistically linked to progressive cognitive decline, comorbid depressive symptoms, and functional disability (57).

In SLE and autoimmune encephalitis, autoreactive CD4+ and CD8+ T cells infiltrate the CNS, targeting anatomically distinct regions—including the prefrontal cortex, hippocampus, basal ganglia, white matter tracts, choroid plexus, and spinal cord—thereby precipitating anxiety, depression, psychosis, and catatonia (58). Collectively, these convergent lines of evidence indicate that T cell subset imbalance, notably Th1/Th17 skewing and Treg deficiency, and the resulting neuroimmune crosstalk constitute a unifying pathophysiological axis underlying the frequent comorbidity between SSc and neuropsychiatric disorders. Gender differences also merit attention. Most studies indicate that women perceive pain more intensely, and pain is a major trigger for anxiety and depression (59). Some studies also suggest that females are more susceptible to anxiety disorders. In contrast, another cross-sectional study found no significant gender differences in the prevalence of anxiety and depressive symptoms (60). Therefore, further investigations are still required in this field.

In the immunopathological overlap between SSc and neuropsychiatric disorders, shared core mechanisms include B-cell immune tolerance breakdown, clonal expansion of autoreactive B cells, and pathogenic autoantibody production. In SSc, dysregulation affects both the composition and functional competence of B-cell subsets. Bregs are reduced in frequency and exhibit impaired IL-10 secretion, which are key deficits that directly compromise peripheral tolerance. Concurrently, aberrantly activated conventional B cells form ectopic aggregates in skin and lung tissues. Within these aggregates, they secrete profibrotic mediators such as IL-6 and TGF-β, present autoantigens to CD4+ T cells, and generate high-affinity, disease-specific autoantibodies. These autoantibodies include antinuclear antibodies (ANA), anti-topoisomerase I antibodies (ATA), anti-centromere antibodies (ACA), and anti-RNA polymerase III antibodies (ARPA), all of which serve as established serological biomarkers and pathogenic effectors in SSc (61). MDD patients display a marked reduction in Bregs and transitional B cells alongside aberrant activation of conventional B-cell compartments. This B-cell dysregulation drives elevated production of proinflammatory cytokines, including IL-6 and TNF-α, thereby amplifying both systemic inflammation and neuroinflammation. Concurrently, serum levels of pathogenic autoantibodies are significantly increased, notably ANA, anti-endothelial cell antibodies (AECA), and antiphospholipid antibodies. These autoantibodies bind to CNS synaptic proteins and neurotransmitter receptors, disrupting synaptic integrity, inducing excitotoxicity and oxidative stress, triggering caspase-dependent neuronal apoptosis, and impairing hippocampal-dependent memory consolidation. These processes are mechanistically linked to core depressive features such as anhedonia, psychomotor slowing, and executive dysfunction (62).

In schizophrenia, N-methyl-D-aspartate receptor (NMDAR) autoantibodies are detected. These autoantibodies can cross the BBB, internalize surface NMDARs, disrupt glutamatergic signaling, and activate microglia and the classical complement pathway, culminating in synaptic pruning, dendritic spine loss, and cortical hypometabolism—neuropathological hallmarks associated with psychosis, cognitive deficits, and functional decline (63). In neuropsychiatric systemic lupus erythematosus (NPSLE), a prototypical model of autoimmune-neuropsychiatric comorbidity, NMDAR autoantibodies are consistently detected. Specifically, in patients with diffuse NPSLE, cerebrospinal fluid (CSF) levels of these autoantibodies are significantly elevated. Upon binding to neuronal surface NMDARs, they trigger glutamatergic excitotoxicity via excessive calcium influx, a pathogenic process that ultimately culminates in neuronal damage and progressive cognitive impairment (64).Epidemiological studies have established a bidirectional association between autoimmune diseases and neuropsychiatric disorders: autoimmune conditions increase the risk of developing neuropsychiatric disorders by 1.43-fold, while pre-existing neuropsychiatric disorders elevate the risk of subsequent autoimmune disease onset by 1.55-fold (65).

In patients with SSc, macrophage phagocytic capacity is markedly impaired, leading to the accumulation of circulating nuclear antigens. Activated macrophages exhibit upregulated expression of chemokines including CCL18, CCL2, and CXCL8, alongside reduced levels of the anti-inflammatory cytokine IL-10. They also infiltrate perivascular regions of the skin, where they contribute to the sustained activation of fibroblasts (66). Within SSc, aberrant activation of M2-polarized macrophages drives progressive tissue and organ fibrosis via the continuous secretion of cytokines (IL-4, IL-13, IL-10) and growth factors (CXCL13, fibronectin, and vascular endothelial growth factor) (67). Notably, increased macrophage abundance and dysregulated activation have also been observed in patients with mental disorders such as MDD, BP, and schizophrenia. This macrophage dysfunction not only perpetuates neuroinflammation but also disrupts microglial function, thereby compromising neuronal survival and synaptic plasticity (68). Activated microglia secrete chemokines (CCL2, CX3CL1) that recruit proinflammatory monocytes and other peripheral immune cells into the central nervous system, further amplifying inflammatory cytokine release through a positive feedback loop (69). Additionally, M1-polarized microglia generate reactive oxygen species (ROS), including nitric oxide (NO), which impair the functional and structural integrity of axons and synapses (70). Collectively, the inflammatory activation profile of macrophages/monocytes—characterized by monocytosis, upregulated inflammatory gene expression, and elevated levels of monocyte/macrophage-derived proinflammatory and anti-inflammatory cytokines in serum/plasma—may represent a key mechanistic link underlying the comorbidity between mental disorders and autoimmune diseases (71).

In SSc, neutrophils exhibit aberrant activation, releasing ROS and proteolytic enzymes to induce oxidative stress. Concurrently, they secrete proinflammatory cytokines (IL-6, TNF-α, IL-1β) and chemokines (CXCL8, CXCL2, CCL2), which amplify and sustain inflammatory cascades. Neutrophil extracellular traps (NETs) not only drive autoimmunity and chronic inflammation but also recruit and activate monocytes, macrophages, and lymphocytes, thereby further exacerbating vascular injury and persistent inflammation. Additionally, neutrophil dysregulation induces the production of anti-neutrophil cytoplasmic antibodies (ANCA), which correlates strongly with elevated risks of ILD and pulmonary embolism (PE) (72). Notably, neutrophil abnormalities also contribute to the pathogenesis of multiple mental disorders. During acute psychotic episodes, activated neutrophils disrupt the blood-brain barrier (BBB), facilitating the translocation of proinflammatory cytokines (TNF-α, IL-6, IL-1β) into the central nervous system (CNS) and accelerating disease progression (73). Elevated NET levels have also been observed in patients with schizophrenia, which may enhance their susceptibility to inflammatory and autoimmune comorbidities (74).

In SSc, the absolute number of natural killer (NK) cells is significantly decreased and exhibits clinically relevant associations. Specifically, SSc patients presenting with arthralgia display a markedly lower NK cell percentage compared to those without joint symptoms. In contrast, anti-centromere antibody (ACA)-positive patients demonstrate elevated NK cell counts, which suggests a potential immunomodulatory role for NK cells in autoantibody production. Moreover, NK cell cytotoxic function is consistently impaired in SSc. Among 48 evaluated patients, 12.5% tested positive for anti-killer cell immunoglobulin-like receptor (KIR) antibodies, a prevalence that substantially exceeds the 3% observed in healthy controls. Furthermore, KIR2DL2, a receptor associated with SSc clinical phenotypes, may dysregulate the anti-fibrotic function of NK cells, promoting the release of cytotoxic mediators that drive cell lysis and cytokine secretion (75). Beyond connective tissue disease, NK cell dysregulation has been increasingly recognized in neuropsychiatric disorders, including tic disorders, schizophrenia, and bipolar disorder (76). During acute psychiatric exacerbations, reduced NK cell cytotoxicity coincides with systemic immune activation, characterized by elevated circulating levels of IL-6, TNF-α, IL-1β, IFN-γ, and IL-8—cytokine alterations that correlate robustly with symptom severity and longitudinal disease progression (77).

Abnormal activation of dendritic cells (DCs) is a consistent feature in SSc with functional consequences extending across immune dysregulation, fibrosis, and vascular injury. DCs in SSc exhibit heightened production of proinflammatory cytokines (IL-6, TNF-α, IL-12, and IFN-α) as well as the platelet-derived chemokine CXCL4. Critically, these mediators drive CD4+ T cell polarization toward Th2 and Th17 lineages, activate monocytes, NK cells, and B cells, and synergistically amplify adaptive and innate immune responses. Moreover, DC-derived signals promote fibroblast-to-myofibroblast transdifferentiation, while CXCL4 directly suppresses endothelial cell proliferation and tube formation. These effects collectively accelerate microvascular rarefaction and obliterative vasculopathy characteristic of SSc (78).

In neuropsychiatric disorders, including schizophrenia and bipolar disorder, DCs serve as key initiators of peripheral immunoinflammatory signaling via toll-like receptors (TLRs). Upon activation, DCs secrete cytokines that breach the BBB, triggering microglial reactivity and sustaining neuroinflammation. This process is mechanistically linked to symptom severity and longitudinal disease course. In parallel, DC-mediated immune crosstalk indirectly perturbs monoaminergic and glutamatergic neurotransmission pathways, providing a plausible immunological basis for core psychiatric manifestations (79).

#### 4.1.2 Innate immunity

A pronounced decline in macrophage phagocytic capacity is observed among SSc patients, giving rise to accumulation of circulating nuclear antigens. Activated macrophages exhibit upregulated expression of chemokines including CCL18, CCL2, and CXCL8, alongside reduced levels of the anti-inflammatory cytokine IL-10. They also infiltrate perivascular regions of the skin, where they contribute to the sustained activation of fibroblasts (66). Within SSc, aberrant activation of M2-polarized macrophages drives progressive tissue and organ fibrosis via the continuous secretion of cytokines (IL-4, IL-13, IL-10) and growth factors (CXCL13, fibronectin, and vascular endothelial growth factor) (67). Notably, increased macrophage abundance and dysregulated activation have also been observed in patients with mental disorders such as MDD, BP, and schizophrenia. This macrophage dysfunction not only perpetuates neuroinflammation but also disrupts microglial function, thereby compromising neuronal survival and synaptic plasticity (68). Activated microglia secrete chemokines (CCL2, CX3CL1) that recruit proinflammatory monocytes and other peripheral immune cells into the central nervous system, further amplifying inflammatory cytokine release through a positive feedback loop (69). Additionally, M1-polarized microglia generate reactive oxygen species (ROS), including nitric oxide (NO), which impair the functional and structural integrity of axons and synapses (70). Collectively, the inflammatory activation profile of macrophages/monocytes—characterized by monocytosis, upregulated inflammatory gene expression, and elevated levels of monocyte/macrophage-derived proinflammatory and anti-inflammatory cytokines in serum/plasma—may represent a key mechanistic link underlying the comorbidity between mental disorders and autoimmune diseases (71).

Neutrophils in systemic sclerosis present abnormal activation and release reactive oxygen species (ROS) as well as proteolytic enzymes, thereby triggering oxidative stress. Concurrently, they secrete proinflammatory cytokines (IL-6, TNF-α, IL-1β) and chemokines (CXCL8, CXCL2, CCL2), which amplify and sustain inflammatory cascades. Neutrophil extracellular traps (NETs) not only drive autoimmunity and chronic inflammation but also recruit and activate monocytes, macrophages, and lymphocytes, thereby further exacerbating vascular injury and persistent inflammation. Additionally, neutrophil dysregulation induces the production of anti-neutrophil cytoplasmic antibodies (ANCA), which correlates strongly with elevated risks of ILD and pulmonary embolism (PE) (72). Notably, neutrophil abnormalities also contribute to the pathogenesis of multiple mental disorders. During acute psychotic episodes, activated neutrophils disrupt the blood-brain barrier (BBB), facilitating the translocation of proinflammatory cytokines (TNF-α, IL-6, IL-1β) into the central nervous system (CNS) and accelerating disease progression (73). Elevated NET levels have also been observed in patients with schizophrenia, which may enhance their susceptibility to inflammatory and autoimmune comorbidities (74).

In SSc, the absolute number of natural killer (NK) cells is significantly decreased and exhibits clinically relevant associations. Specifically, SSc patients presenting with arthralgia display a markedly lower NK cell percentage compared to those without joint symptoms. In contrast, anti-centromere antibody (ACA)-positive patients demonstrate elevated NK cell counts, which suggests a potential immunomodulatory role for NK cells in autoantibody production. Moreover, NK cell cytotoxic function is consistently impaired in SSc. Among 48 evaluated patients, 12.5% tested positive for anti-killer cell immunoglobulin-like receptor (KIR) antibodies, a prevalence that substantially exceeds the 3% observed in healthy controls. Furthermore, KIR2DL2, a receptor associated with SSc clinical phenotypes, may dysregulate the anti-fibrotic function of NK cells, promoting the release of cytotoxic mediators that drive cell lysis and cytokine secretion (75). Beyond connective tissue disease, NK cell dysregulation has been increasingly recognized in neuropsychiatric disorders, including tic disorders, schizophrenia, and bipolar disorder (76). During acute psychiatric exacerbations, reduced NK cell cytotoxicity coincides with systemic immune activation, characterized by elevated circulating levels of IL-6, TNF-α, IL-1β, IFN-γ, and IL-8—cytokine alterations that correlate robustly with symptom severity and longitudinal disease progression (77).

Abnormal activation of dendritic cells (DCs) is a consistent feature in SSc with functional consequences extending across immune dysregulation, fibrosis, and vascular injury. DCs in SSc exhibit heightened production of proinflammatory cytokines (IL-6, TNF-α, IL-12, and IFN-α) as well as the platelet-derived chemokine CXCL4. Critically, these mediators drive CD4+ T cell polarization toward Th2 and Th17 lineages, activate monocytes, NK cells, and B cells, and synergistically amplify adaptive and innate immune responses. Moreover, DC-derived signals promote fibroblast-to-myofibroblast transdifferentiation, while CXCL4 directly suppresses endothelial cell proliferation and tube formation. These effects collectively accelerate microvascular rarefaction and obliterative vasculopathy characteristic of SSc (78).

In neuropsychiatric disorders, including schizophrenia and bipolar disorder, DCs serve as key initiators of peripheral immunoinflammatory signaling via toll-like receptors (TLRs). Upon activation, DCs secrete cytokines that breach the BBB, triggering microglial reactivity and sustaining neuroinflammation. This process is mechanistically linked to symptom severity and longitudinal disease course. In parallel, DC-mediated immune crosstalk indirectly perturbs monoaminergic and glutamatergic neurotransmission pathways, providing a plausible immunological basis for core psychiatric manifestations (79).

### 4.2 Microbiota-gut-brain axis dysregulation

The gut microbiota (GM) serves as a pivotal modulator of immune homeostasis, and its compositional and functional dysbiosis is mechanistically linked to the initiation, progression, and systemic manifestations of SSc. Critically, GM-derived short-chain fatty acids (SCFAs) promote the differentiation and suppressive function of Tregs while inhibiting the expansion and pathogenicity of T helper 17 (Th17) cells, thereby reinforcing peripheral immune tolerance. In rheumatoid arthritis, for example, dysbiosis characterized by enrichment of pro-inflammatory taxa and depletion of immunoregulatory commensals drives chronic systemic inflammation, autoantibody production—including rheumatoid factor (RF) and anti-cyclic citrullinated peptide (anti-CCP) antibodies—and aberrant activation of both T and B lymphocyte compartments (80). Notably, SSc and neuropsychiatric disorders exhibit convergent dysbiotic signatures, including reduced microbial alpha diversity, diminished abundance of SCFA-producing Clostridia clusters, and increased colonization by mucosa-associated pathobionts (81). This dysbiosis compromises intestinal epithelial barrier integrity to induce a “leaky gut” phenotype and activates innate immune sensors, resulting in elevated circulating levels of IL-6, TNF-α, and IL-1β. These cytokines propagate neuroinflammation via three non-mutually exclusive pathways: (i) modulation of brain glutamine metabolism through the gut–liver–brain axis; (ii) vagus nerve-mediated afferent signaling to brainstem nuclei such as the nucleus tractus solitarius; and (iii) disruption of the BBB, which facilitates the translocation of cytokines and bacterial lipopolysaccharide (LPS) into the CNS parenchyma. Within the brain, these mediators bind neuronal and microglial pattern recognition receptors, downregulating tryptophan hydroxylase 2 (TPH2) and tyrosine hydroxylase (TH), suppressing serotonin and dopamine synthesis, impairing synaptic vesicle recycling, and reducing dendritic spine density. These effects collectively contribute to neurobehavioral deficits including anhedonia, psychomotor retardation, and working memory impairment (82, 83).

Collectively, these findings position GM-driven dysregulation of the microbiota–gut–brain axis not merely as a correlative feature but as a pathogenically active interface. Through this interface, peripheral immune dysfunction in SSc may directly contribute to the development of neuropsychiatric manifestations.

### 4.3 Vascular pathologies and cerebral functional impairment

Vascular dysfunction is a hallmark pathological feature of SSc. Sustained release of pro-inflammatory mediators, including IL-17, IL-6, and granulocyte-macrophage colony-stimulating factor (GM-CSF), exacerbates endothelial dysfunction, oxidative stress, and microvascular endothelial cell apoptosis. When such vascular pathologies extend to the CNS, they disrupt cerebral hemodynamics, manifesting as chronic hypoperfusion, vasoconstriction, and impaired neurovascular coupling. Over time, these perturbations drive cortical thinning, gray matter volume loss, and regional neural circuit dysfunction, which collectively contribute to the development of neuropsychiatric symptoms including depression, fatigue, and cognitive decline (9, 84). Oxidative stress serves as a central driver of SSc-related vascular damage. Aberrant activation of NADPH oxidase and mitochondrial dysfunction synergistically promote excessive reactive oxygen species (ROS) production, which compromises the structural and functional integrity of the BBB (85). Increased BBB permeability facilitates the transmigration of peripheral immune cells and pro-inflammatory cytokines (IL-1β, TNF-α, and IL-6) into the CNS parenchyma, establishing a persistent neuroinflammatory microenvironment. Notably, sustained neuroinflammation significantly downregulates the expression of neurotrophic factors, most prominently brain-derived neurotrophic factor (BDNF). These factors are critical for neuronal survival, synaptic plasticity, and neurogenesis. This suppression of neurotrophic signaling impairs endogenous neural repair and regeneration processes and represents a key mechanistic link between SSc-related vascular pathology and comorbid depressive symptoms (86).

### 4.4 Hypothalamic-pituitary-adrenal axis dysregulation

Dysregulation of the HPA axis represents a shared pathophysiological cornerstone in SSc and comorbid neuropsychiatric disorders. Chronic exposure to physical symptom burden, including pain, skin fibrosis, and gastrointestinal dysmotility, as well as psychosocial stressors in SSc patients drives persistent HPA axis activation that culminates in elevated circulating glucocorticoid levels. In early disease stages, hypercortisolemia exerts paradoxical immunomodulatory effects: while suppressing some immune functions, it may simultaneously prime monocytes and macrophages for enhanced production of pro-inflammatory cytokines. With prolonged activation, however, the HPA axis undergoes adaptive exhaustion, leading to functional glucocorticoid resistance despite variable systemic cortisol concentrations (87). These alterations attenuate cortisol’s anti-inflammatory and immunosuppressive bioactivity, precipitating chronic immune dysregulation. Concurrently, they trigger sustained neuroinflammation that impairs hippocampal and prefrontal cortical neuroplasticity, disrupts synaptic transmission, and reduces adult neurogenesis, forming the neurobiological substrate for depression, anxiety, and cognitive dysfunction. These findings highlight the HPA–immune–neural axis as a critical mechanistic nexus linking autoimmune pathogenesis to neuropsychiatric comorbidity (87, 88).

As previously noted, interstitial lung disease is a prevalent complication of SSc, and pulmonary fibrosis commonly develops in affected patients. Studies have shown a high incidence of anxiety, depression and stress among SSc patients with pulmonary fibrosis, and anxiety is the predominant symptom. Long-term oxygen supplementation and persistent dyspnea induce negative moods, while anxiety and depression amplify physical distress and compromise treatment adherence (42). Cutaneous fibrosis causes morphological changes and dysfunction in SSc patients, limiting their daily activities and deteriorating quality of life, which may contribute to the onset of depression and other emotional disorders. However, a direct association between fibrosis and comorbid mental disorders in SSc has not been verified by existing studies, and larger sample data are required for further exploration (5).

## 5 Therapeutic implications and future perspectives

Current therapeutic strategies for SSc follow a tiered management principle. For patients with mild disease, treatment primarily combines non-pharmacological interventions (including rehabilitation and functional training, psychosocial support, etc.) with single-agent symptomatic therapy. In cases with multi-organ involvement, combined regimens of immunosuppressants and targeted agents are adopted. For end-stage and high-risk patients, intensive immunosuppressive therapy plus hematopoietic stem cell transplantation is recommended.

Vascular manifestations such as Raynaud’s phenomenon and digital ulcers are treated with vasoactive agents including calcium channel blockers and iloprost; monotherapy is applied for mild conditions, while combination therapy is used for severe cases. Mycophenolate mofetil (MMF), rituximab and tocilizumab exert therapeutic effects on both cutaneous and pulmonary fibrosis. Nintedanib specifically targets pulmonary fibrosis and can be combined with immunosuppressants to enhance efficacy. High-dose glucocorticoid monotherapy is not recommended for SSc; only short-term low-dose glucocorticoids may be used as adjuvant treatment to avoid triggering scleroderma renal crisis.

Considering the high prevalence of comorbid depression and anxiety among Sc patients, routine psychological assessment using scales such as Depression, Anxiety and Stress Scale – 21(DASS-21) and HADS is advised. Psychiatric medications should be prescribed in a standardized manner in collaboration with psychiatrists when necessary (89).

Given the bidirectional interplay between systemic autoimmunity and neuropsychiatric impairment, current therapeutic strategies for neuropsychiatric comorbidities in SSc encompass three mechanistically distinct yet synergistic domains, as summarized in **Table 2**: immunomodulation, psychological intervention, and neuromodulation. Emerging targeted immunomodulators, including JAK inhibitors (tofacitinib, baricitinib) and IL-6 receptor antagonists (tocilizumab, sarilumab), exert dual anti-inflammatory effects by suppressing CNS JAK-STAT-mediated glial activation and attenuating peripheral IL-6-driven cytokine cascades, thus alleviating depressive symptoms, fatigue, and anhedonia in preclinical and early clinical trials. In parallel, cognitive behavioral therapy (CBT) effectively reduces pain interference and depressive severity in SSc patients, with benefits mediated partly by enhanced illness self-efficacy and maladaptive cognitive restructuring. Similarly, biofeedback-assisted autonomic training restores parasympathetic tone, lowering heart rate variability markers of sympathetic dominance and state anxiety. For non-invasive neuromodulation, approaches such as dorsolateral prefrontal cortex (DLPFC)-targeted transcranial direct current stimulation (tDCS) modulate cortical excitability to relieve chronic pain and reverse negative attentional bias. Furthermore, both invasive and non-invasive vagus nerve stimulation (VNS) exert anti-inflammatory effects via the cholinergic anti-inflammatory pathway, dampen amygdala hyperactivity, and reduce fibroblast activation, concurrently mitigating anxiety, neuroinflammation, and cutaneous fibrosis.

**Table 2:** Summary of treatment strategies for psychiatric comorbidities in systemic sclerosis.

Treatment category	Specific methods/techniques	Mechanism of action/therapeutic objective	Reference
Psychobehavioral Intervention	CBT	Long-term improvement in pain intensity, self-efficacy and depressive symptoms	(90)
	Biofeedback Training	Regulates autonomic nervous function and alleviates anxiety.	(91)
Targeted Immunomodulatory Psychopharmacological Intervention	JAK inhibitors (e.g., Tofacitinib)	Tofacitinib inhibits hippocampal JAK-STAT signaling, reduces microglia-mediated neuroinflammation and elevates BDNF levels, exerting antidepressant effects in mouse models of depression.	(92, 93)
	IL-6 receptor antagonist (e.g., tocilizumab)	Blocks the IL-6 signaling pathway, alleviates systemic inflammation, and improves fatigue and emotional symptoms.	
Neuromodulation techniques	tDCS	significantly relieve chronic pain, improve depressive mood and negative cognition.	(94)
	VNS	Inhibits the release of pro-inflammatory factors such as TNF-α, modulates brain regions involved in emotional regulation, and alleviates fibrosis and anxiety symptoms.	(95)

CBT, Cognitive Behavioral Therapy; JAK, Janus kinase; tDCS, Transcranial Direct Current Stimulation; VNS, Vagus Nerve Stimulation.

Addressing psychiatric comorbidities in SSc represents both a clinical imperative and a scientifically strategic opportunity. Clinically, this focus aims to mitigate overall disease burden, improve health-related quality of life, and optimize long-term patient outcomes; scientifically, it enables the elucidation of shared pathophysiological pathways at the immune–neural interface. Emerging evidence supports a critical paradigm shift toward integrated, mechanism-informed interventions.

Combining immunomodulatory agents with precision psychological support and neuroprotective approaches may yield synergistic effects by targeting overlapping biological substrates, thereby allowing concurrent modulation of both SSc disease progression and associated neuropsychiatric manifestations. This integrative framework holds substantial translational promise, not only for the management of SSc but also as a scalable model for other autoimmune disorders complicated by comorbid mental illness.
